# Amblyopia among Patients Attending the Outpatient Department of Ophthalmology of a Tertiary Care Centre: A Descriptive Cross-sectional Study

**DOI:** 10.31729/jnma.7868

**Published:** 2022-10-31

**Authors:** Sikshya Adhikari, Rinkal Suwal, Basanta Singh, Rashmi Shrestha, Sudip Karki, Bijay Khatri

**Affiliations:** 1 Department of Optometry, B.P. Eye Foundation, Hospital for Children, Eye, ENT, and Rehabilitation Services, Madhyapur Thimi, Bhaktapur, Nepal; 2 Academic and Research Department, B.P. Eye Foundation, Hospital for Children, Eye, ENT, and Rehabilitation Services, Madhyapur Thimi, Bhaktapur, Nepal

**Keywords:** *amblyopia*, *anisometropia*, *astigmatism*, *refractive errors*

## Abstract

**Introduction:**

Amblyopia is defined as a reduction in visual acuity unilaterally or bilaterally without any detectable cause. It is a major public health issue in developing and underdeveloped countries. Its prevalence is usually underestimated because of proper study and lack of awareness. The aim of the study was to find out the prevalence of amblyopia among patients attending the Outpatient Department of Ophthalmology of a tertiary care centre.

**Methods:**

This descriptive cross-sectional study was conducted among outpatients visiting a tertiary care centre in the Outpatient Department of Ophthalmology between 1 January 2017 to 31 December 2019. Ethical approval was obtained from the Ethical Review Board (Registration number: 407/2020 P). All patients had gone through a comprehensive eye examination. Convenience sampling was used. Point estimate and 99% Confidence Interval were calculated.

**Results:**

Among 82972 patients, prevalence of amblyopia was 344 (0.41%) (0.37-0.46, 99% Confidence Interval). Amblyopia was more common in anisometropia 263 (63.50%). A total of 117 (34%) patients had no history of eye examination and were newly diagnosed with amblyopia. Astigmatism was the most common type of refractive error among 224 (56.70%) amblyopic patients.

**Conclusions:**

The prevalence of amblyopia was found to be lower than in previous studies conducted in similar settings. Early detection and diagnosis of amblyopia can help to design more effective plans and treatments to reduce amblyopia through optical correction and amblyopia therapy.

## INTRODUCTION

Amblyopia is defined as a reduction in visual acuity (VA) unilaterally or bilaterally without any detectable cause, and is the common cause of monocular visual impairment (VI) in children, young and middle-aged adults.^1,2^ Etiologies include high refractive errors, anisometropia, strabismus, ocular media opacity, deprivation in the optical axis, and combination of these etiologies. Wide variations of amblyopia from 0.20% to 5.30% have been reported. However, it depends upon the population density and assessment method.^3,4^

Early screening and appropriate treatment for amblyopia can decrease the prevalence belonging to amblyopia.^1^ Still, studies have found good improvement in VA and binocular function at any age.^5^ To our knowledge, there has been no study regarding the prevalence of amblyopia in all age groups in Nepal.

The aim of the study was to find out the prevalence of amblyopia among patients attending Outpatient Department of Ophthalmology of a tertiary care centre.

## METHODS

This descriptive cross-sectional study was conducted at B.P. Eye Foundation, Hospital for Children, ENT and Rehabilitation Services (CHEERS). A medical record review of pateints from 1 January 2017 to 31 December 2019 was done, who were diagnosed with amblyopia at the tertiary eye care centre. Ethical approval was obtained from the Ethical Review Board of Nepal Health Research Council (Registration number: 407/2020 P). All the patients with age ≥5 years presenting to the outpatient department (OPD) of ophthalmology of CHEERS hospital were included. Patients having a history of ocular surgery for congenital cataracts and ptosis which are suspected cases of amblyogenic factors were also included. Patients with a history of trauma and any ocular pathology affecting refraction and VA were excluded. Convenience sampling was used. The sample size was calculated by using the following formula:


$$\begin{array}{ll} \text{n} & ={\text{Z}}^{2}\times \frac{\text{p}\times \text{q}}{{\text{e}}^{\text{2}}}\\ & =2.576^{2}\times \frac{0.50\times 0.50}{0.01^{2}}\\ & =16590 \end{array}$$

Where,

n= minimum required sample size
Z= 2.576 at 99% Confidence Interval (CI)
p= prevalence taken as 50% for maximum sample size calculation
q= 1-p
e= margin of error, 1%

The calculated sample size was 16590. After quadrupling the sample size, the sample size was 66,360. However, a total of 82,972 sample size was taken. All subjects had gone through a comprehensive eye examination including history taking, vision, refraction with streak retinoscope, ocular alignment and motility, anterior segment and dilated fundus examination with slit lamp bio-microscope with 90 Diopter (D) lens.

Snellen chart was used for VA assessment in both children and adults group. Snellen chart at 6 meters of distance was carried out for VA assessment of adult patients and illiterate patients were instructed using tumbling E chart. Both uncorrected visual acuity (UCVA) along with best-corrected visual acuity (BCVA) were recorded. Cycloplegic refraction was done in all patients in the children group with 1% cyclopentolate, 1% tropicamide and 1% cyclopentolate (CTC), which was installed in each eye 3 times within 15 minutes of interval. Then, after the 15 minutes of the last drop installation retinoscopy was performed. In cases for children, prescribed glasses were again noted in one month and a re-examination was done. If BCVA was 6/12 or worse, then the subjects were diagnosed with amblyopia and incorporated into the analysis.^6^ A cover test was performed both at a distance of 6 meters and near 40 cm with and without correction to see the ocular alignment. The subject underwent a detailed orthoptics evaluation if any kind of ocular misalignment was noted. The prism bar cover test (PBCT) was used for quantifying the deviation.

Ambylopia was classified as unilateral and bilateral associated visual defect ametropic, anisometropic, strabismic, mixed and stimulus deprivation were also assessed.^6^ Refractive errors were categorized into myopia, hypermetropia and astigmatism. Astigmatism was further classified as simple myopic or hyperopic, compound myopic or hypermetropic and mixed and also grouped as with the rule (WTR), against the rule (ATR), and oblique. Further severity of amblyopia was classified as mild, moderate and severe on the basis of BCVA.^7^

Data were entered in an excel sheet and analysed with IBM SPSS Statistics 26.0. Point estimate and 99% CI were calculated.

## RESULTS

Out of 82,972 new eye patients, amblyopia was found in 344 (0.41%) (0.37-0.46, 99% CI). Among them, there were 135 (39.20%) children and 209 (60.80%) were adults. A total of 227 (65.98%) patients were already diagnosed with amblyopia and 117 (35.01%) patients were newly diagnosed (Table 1).

**Table 1:** Baseline characteristics (n= 344).

Characteristics	n (%)
**Age-group (years)**	
5-15	135 (39.24)
16-60	209 (60.75)
**Sex**	
Male	206 (59.88)
Female	138 (40.11)
**History of using glasses**	
Yes	125 (36.33)
No	102 (29.65)
Newly diagnosed	117 (34.01)
**Diagnosis of amblyopia**	
Newly diagnosed	117 (34.01)
Previously diagnosed	227 (65.98)
**Year of diagnosis**	
2017	104 (30.23)
2018	119 (34.59)
2019	121 (35.17)
**Type**	
Unilateral amblyopia	274 (79.65)
Bilateral amblyopia	70 (20.34)

Total amblyopic eyes were 414 (63.52%) among amblyopic patients. Anisometropic amblyopia was found in 263 (78.74%) eyes. Significant refractive error was found in 395 (95.41%) eyes. Astigmatism was found in 224 (54.10%) followed by hyperopia in 137 (33.09%) and myopia in 34 (8.21%). Commonest type of astigmatism were rule astigmatism seen in 154 (68.75%) and compound myopic seen in 86 (38.39%). A total of 183 (44.20%) amblyopic eyes were classified as having moderate amblyopia, 142 (34.29%) with severe amblyopia and 89 (21.49%) with mild amblyopia.

**Table 2:** Coexisting visual abnormalities in eyes of patients with amblyopia (n= 414).

Amblyopia	Right eye n (%)	Left eye n (%)	Total n (%)
Anisometropic	125 (30.19)	138 (33.33)	263 (63.52)
Strabismic	6 (1.44)	14 (3.38)	20 (4.83)
Ametropic	52 (12.57)	58 (14)	110 (26.57)
Mixed (Strabismic/anisometropia)	4 (0.96)	7 (1.69)	11 (2.65)
Secondary to nystagmus	3 (0.72)	3 (0.72)	6 (1.44)
Stimulus deprivation	3 (0.72)	1 (0.24)	4 (0.96)

## DISCUSSION

Overall, the prevalence of amblyopia in our study was 0.41%. Among that, 0.78% were children and 0.32% were adults. This is the lower prevalence in comparison to the previous other study ranging from 0.80% to 3% depending on the definition used, frequency of visual screening programs and population studied.^2,7-12^ It might be due to the school screening programs in recent years, increasing knowledge about amblyopia, refractive errors, the importance of routine eye checkups among the parents, and better public education.

In our study, amblyopia was more common in males than females. A study also found similar gender differences among the amblyopic eye.^7^ An explanation for this gender difference might be because of the fact that fewer females are seen in hospital-based setups compared to males in our patriarchal society.

Refractive error was the leading cause of amblyopia in our study. A large number of the amblyopic eyes was astigmatism, i.e, 224 (56.70%). Different studies done previously in Nepal and abroad have also depicted astigmatism to be the main amblyogenic factor.^7,13,14^ With the rule astigmatism was a common type of astigmatism, i.e, 154 (68.80%) similar to other studies.^7^ Therefore, astigmatism might be a main amblyogenic factor for amblyopia. Secondly, hyperopia was the second most common cause of amblyopia and myopia to be least common in amblyopia. Surprisingly, compound myopic astigmatism was the most common type of astigmatism. In contrast, another study has found compound hyperopic astigmatism to be the commonest type.^7^ Moreover, the type of refractive error which could be the main amblyogenic factor might be a further research interest.

Anisometropic amblyopia was the common cause of amblyopia in our study presenting in 269 (63.50%) patients with amblyopia, which was similar to the findings of other studies.^7^ On the contrary, strabismic amblyopia was most common in another study.^15^ However, in our study, strabismic amblyopia was found only in 20 (4.80%) patients with amblyopia.

We have also included amblyopia secondary to nystagmus which has not been yet reported in different amblyopia prevalence studies in Nepal. However, amblyopia secondary to nystagmus was the least common. Yet, it might be due to lower prevalence of nystagmus.^16^ In nystagmus, there is a higher chance to develop amblyopia which may lead to low vision.^17^ Therefore, nystagmus being a less common involvement in amblyopia might be a major amblyogenic factor whenever there is nystagmus.

Studies regarding the prevalence of amblyopia are mainly focused on children as early diagnosis is important for the treatment of amblyopia.^7,9-12^ However, adults from developing countries like Nepal might not be aware of their condition. Also in our study, out of a total of 344 patients, 117 (34%) patients were newly diagnosed with amblyopia. This shows the further need for awareness of amblyopia. Still, there are difficulties to evaluate the prevalence of amblyopia in elderly populations. Various eye diseases such as age-related macular degeneration or cataract may mask amblyopia in the elder age group,^18^ and this might rise difficulties in the distribution of the cause of amblyopia. Amblyopia is a common cause of the ongoing burden of visual impairment among the older population.^19^ Therefore, regarding all these aspects, we have included the prevalence of amblyopia in adults similar to previous studies.^2,14,19^

Our study highlights the need for an awareness program of amblyopia in developing countries like Nepal, both in communities and hospital-based. Conducting preschool and school screening programs in children would be effective to find out the different amblyogenic factors like refractive error and strabismus. This will help for the early detection and diagnosis of amblyopia through which we can design more effective plans and treatments to reduce amblyopia through optical correction and amblyopia therapy.

The present study was conducted in a tertiary-level hospital in Bhaktapur. It is a single-centre study in an urban location and the prevalence of amblyopia in this study cannot be generalized to the larger population.

## CONCLUSIONS

The prevalence of amblyopia was found to be lower than in previous studies conducted in similar settings. It might be due to school screening programs in recent years, increasing knowledge about amblyopia, refractive errors, the importance of routine eye checkups among the parents, and better public education. Early detection and diagnosis of amblyopia can help to design more effective plans and treatments to reduce amblyopia through optical correction and amblyopia therapy.
